# Incorrect ICBM-DTI-81 atlas orientation and white matter labels

**DOI:** 10.3389/fnins.2013.00004

**Published:** 2013-01-25

**Authors:** T. Rohlfing

**Affiliations:** SRI International, Neuroscience Program, Center for Health SciencesMenlo Park, CA, USA

## 1. Introduction

The ICBM-DTI-81 atlas (Mori et al., 2008; Oishi et al., 2008)^1^ provides perhaps the most widely used reference coordinate system for group analyses of diffusion tensor MR images. The atlas comprises, among other data, structural images (e.g., *T*_2_-weighted), scalar maps of diffusion (e.g., fractional anisotropy), and a map of manually labeled major white matter fiber tracts.

Unfortunately, in our work using this atlas, we have encountered several substantial problems in terms of (a) correctness of spatial orientation and (b) correctness of white matter tract labels. These issues raise questions about the validity of published studies using the ICBM-DTI-81 atlas. We also detected substantial changes applied to the atlas files with no documentation or user notification. Because the changed files are distributed under the same name and the earlier versions are no longer available, it is virtually impossible to reproduce published research using the atlas.

We have contacted the atlas maintainers with our concerns and have been in active communication with them since to discuss possible fixes. As correcting the atlas turned out to take considerable time, however, and because it is not feasible to notify all users of the atlas directly, we feel that communicating our findings to the neuroimaging community at large is warranted. The atlas maintainers were apprised of our intent to write and publish this article and did not raise any objections. Thus, researchers using the ICBM-DTI-81 atlas can verify the validity of their analyses. To this end, we provide a discussion of the significance and potential impact of each problem identified, as well as detailed recommendations for confirming whether or not a particular study was affected.

## 2. Materials

We first downloaded the ICBM-DTI-81 on August 4, 2010 and refer to this version, which is archived on our local servers but no longer available for download, as the “2010 ICBM-DTI-81.” We downloaded the atlas again on August 28, 2012 and refer to this version as the “2012 ICBM-DTI-81.” The key atlas files contained in the 2010 ZIP archives are time stamped November 5, 2009. In the files downloaded in 2012, the time stamps for white matter label map and label lookup file (but not structural images or README file) have changed to February 15, 2011.

Each time, we downloaded the atlas images in NIfTI file format^2^, rather than “raw” format, because the NIfTI file header contains a well-defined specification of the spatial relationship between the image pixels and the major directions of subject anatomy (i.e., left/right, anterior/posterior, and inferior/superior), but the most significant issues pointed out herein (e.g., mislabeled regions; undocumented revisions) affect the NIfTI and raw image files equally.

For comparison, we obtained the “JHU” *T*_2_-weighted template image and white matter atlas distributed with FSL 4.1.9^3^ (Smith et al., 2004), which is an earlier version of the ICBM-DTI-81 atlas. We also obtained the *T*_2_-weighted “MNI152” template included with SPM8^4^, as it defines the coordinate space used for creating the JHU and ICBM-DTI-81 atlases (Mori et al., 2008).

## 3. Results

### 3.1. Consistency between atlases

**Figure 1:**
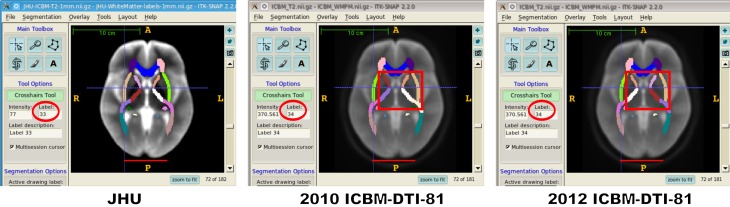
**Display of the same axial slice (*z* = 0 mm) from the *T*_2_-weighted JHU template, and the 2010 (center) and 2012 (right) ICBM-DTI-81 atlases (screen shots from ITK-SNAP 2.2.0).** Horizontal red lines highlight the increased posterior protrusion of the occipital pole of the left hemisphere in the JHU template, which is consistent with known asymmetry of the brain in right-handed individuals (LeMay and Kido, 1978). For comparison of white matter labels, color-coded overlays have been added to the structural images (all label maps used the same color lookup table). The cross-hair cursor points to the same point in the right brain hemisphere (according to image coordinates) in all three atlases. The label under the cursor (circled in red) correctly denotes “EC-R” (#33) in the JHU label map, but “EC-L” (#34) in the 2010 ICBM-DTI-81 label map. In the 2012 ICBM-DTI-81 label map, label #34 now corresponds to “EC-R” due to changed label lookup table. Note also the relabeling of previously mislabeled regions in the brain center, apparent from changed region colors inside the red box.

We first demonstrate that the spatial orientation of the anatomy in the distributed ICBM-DTI-81 atlas is left/right mirrored with respect to the JHU template (also for the MNI152 image; not shown). To this end, Figure 1 shows screen shots of the *T*_2_-weighted template images made using ITK-SNAP^5^ (Yushkevich et al., 2006). We confirmed the same orientation mismatch using three different other NIfTI reader implementation: MRIcron^6^, CMTK 2.2.0^7^, and 3D Slicer 4.1.1^8^.

It is telling that the order in which the image pixels are stored is identical in the atlas files from all four different sources, which we confirmed using FSL's “fslhd” tool. In short, each pixel row is stored in the patient right–left direction, but NIfTI's coordinate space is defined such that the *x* coordinate increases *from* patient left *to* patient right. Thus, the pixels in each image row are stored in the opposite direction, and the space direction vector for the pixel index needs to have a negative sign. This is the case for the JHU image, but not the ICBM-DTI-81 files. In other words, simply changing the sign of the direction vector from + to − would bring the ICBM-DTI-81 images into the correct orientation.

### 3.2. Consistency with white matter labels

Second, we confirmed whether the region labels in the white matter parcellation were on the correct side of the image coordinate space. For all unilateral regions, the pixels labeled as left-hemisphere regions should be located on the subject-left side of the image anatomical coordinate space, and the right-hemisphere regions on the other.

As Figure 1 shows, this is the case for the JHU but not the 2010 ICBM-DTI-81 atlas. For the 2012 atlas, the region labels are *formally* in the correct hemisphere, but not because the label image orientation has been fixed. Instead, the label lookup table has been edited to assign each unilateral region label to the corresponding region in the opposite hemisphere. In other words, the image coordinate system is still incorrect, but by making the label lookup table equally incorrect, both hemisphere swaps cancel each other out. As a side effect, while the label lookup table of the 2010 atlas was mostly consistent with the JHU atlas label lookup table, the 2012 lookup table is now inconsistent for all bilateral regions.

What is even more concerning, however, is that 15 regions in the 2010 ICBM-DTI-81 white matter label map were mislabeled altogether. This is apparent from the switching of region colors in the central brain, highlighted in Figure 1 by red boxes. The exact nature of the incorrect label assignment, and the relabeling applied to “fix” it, can be summarized as follows: region #7 (“CST-R”) changed to #21 (“RLIC-L”); region #21 (“RLIC-R”) changed to #20 (“PLIC-R”); all labels in the range from #8 to #20 were decreased by one (the labels in this range labeled the correct structure in the 2010 atlas, but in the opposite hemisphere of the remaining labels in the 2010 label map).

## 4. Discussion

### 4.1. Summary

To summarize, we have identified the following problems with the ICBM-DTI-81 atlas. First and foremost, we found that substantial changes were made to the atlas without version control, documentation, or user notification.

In the 2010 version of the ICBM-DTI-81 atlas, the following flaws were present:

1. Anatomy likely mirrored in the left–right anatomical direction.
2. Majority of unilateral white matter labels on the wrong side of the image coordinate system.
3. Fifteen white matter regions mislabeled altogether.
4. Four labels (IFO-L/R and UNC-L/R) in conflict with labels in JHU atlas.

The following flaws either persist in the current, 2012 version of the ICBM-DTI-81 atlas, or were newly introduced:

1. Anatomy likely mirrored in the left–right anatomical direction.
2. All unilateral labels now on the correct side of the image coordinate system, but represent anatomy that truly belongs on the opposite side.
3. All unilateral labels use opposite index values with respect to JHU atlas.

Without going back to the original 152 subjects used to create the MNI152 template space, we cannot confirm with certainty whether the anatomy is mirrored in the ICBM-DTI-81 atlas with respect to the actual anatomy, or mirrored in the JHU and MNI152 files. However, it should be noted that the JHU and MNI152 templates are consistent with known asymmetry of the brain, specifically the posterior protrusion of the left occipital pole in right-handed individuals (LeMay and Kido, 1978), whereas the ICBM-DTI-81 template is not. Also, the agreement of JHU and MNI152 templates with one another makes it likely that they are correct and the ICBM-DTI-81 files incorrect.

This point of view is further supported by the observation that in the 2010 atlas, those labels that actually corresponded to the correct white matter structure in general unanimously mapped to the opposite hemisphere as would be expected based on the NIfTI image coordinates. Thus, by simply inverting the direction of the *x* coordinate in the NIfTI header, the 2010 atlas files would both match the anatomy shown in the *T*_2_ images and put the correct labeled regions on the correct side of the anatomical coordinate system. Occam's razor thus compels us to favor the hypothesis that the ICBM-DTI-81 image coordinate system is simply reversed in the left–right direction.

### 4.2. Significance

The various flaws of the ICBM-DTI-81 atlas vary greatly in their potential impact. Using a left–right mirrored template likely has a small effect on the accuracy of spatial normalization, because the two brain hemispheres are almost, but not entirely, symmetric. Thus, aligning a left-hand-side subject hemisphere with a left template hemisphere should be slightly more accurate than aligning it with a right template hemisphere (analogous for the right-hand-side subject hemisphere).

One would, therefore, expect studies that use a correctly oriented template to be slightly more accurate than studies using a mirrored template, at least if we assume that the subject images themselves are oriented correctly. These errors have serious implications for studies claiming “laterality” effects.

Mislabeled white matter structures are a more serious problem. In the 2010 atlas, some region labels were not only switched between the left and right hemispheres but were labeling different structures altogether. Studies using the older atlas are therefore likely affected. Unlike the problem of incorrect image orientation, the mislabeling problem affects the atlas files distributed in both NIfTI and raw format, because no image header could have corrected the label-to-region assignments.

Using region labels that are inconsistent with other distributions of the same atlas is more of a nuisance than a serious problem, but it would have been simple enough to avoid this nuisance. What does create potential problems, however, is that by fixing the region labels rather than the orientation of the template images, *all* unilateral labels in the 2012 atlas version now refer to the wrong hemisphere when the images are read in anatomically correct orientation.

### 4.3. Recommendations

As a first step to confirm the validity of their results, researchers whose studies might be affected should carefully inspect the white matter region labels in the image files that they used to ensure that all labels correspond to the correct structures. Second, it should be confirmed that when subject images are aligned with the template, the correct hemispheres are being aligned. If that is not the case due to the incorrect template coordinates, then the template coordinate system should be fixed to the anatomically correct orientation, and left and right hemisphere labels should be re-assigned to match the correct anatomy.

Technically, the simplest way to fix the laterality mismatch of ICBM-DTI-81 with typical brain anatomy and to match the orientation of the MNI152 template space would be to adjust the orientation information of the NIfTI image headers. Then, the exchange of left- and right-hemisphere label IDs that occurred between 2010 and 2012 would no longer be needed and could be reversed. This would restore much of the consistency with the JHU atlas.

For current users of the atlas it would further be desirable to be able to distinguish the newly corrected from previous atlas versions. This could be easily achieved by assigning an explicit, distinct revision identifier to the corrected version. Should additional corrections be needed in the future, or additions or improvements made to the atlas, then such revision labeling would provide for a straight-forward method of identifying in publications which specific atlas version was used in the study. Finally, as is established practice in software development for example, a change log document distributed with each new atlas version could detail the differences between the new and the previous revision of the data set, which would greatly assist its users in determining whether their previous analyses may need to be revisited.

## 5. Conclusion

We have identified, characterized, and documented several flaws in the ICBM-DTI-81 atlas as distributed until very recently. Mistakes do happen, and with ever increasing efforts to share research data, incorrect data will invariably find its way into the community on occasion. It is thus not the intent of this article to single out or criticize the maintainers of the ICBM-DTI-81 atlas, who are currently preparing a corrected atlas for release.

When an error is discovered in a shared data set, however, then best practices of data sharing should include a prompt, complete, and proper correction. The corrected data should be explicitly identified as a new revision, and the change applied to the previous version should be documented to facilitate gauging its impact on earlier studies. Ideally, users of the previous data set should finally be informed of the new version, which could be achieved by personal communication (if data access required user registration), forum postings (e.g., on a portal such as NITRC^9^), a note in the atlas distribution system, or an article such as this.

## Footnotes

^1^Available from https://www.loni.ucla.edu/Atlases/Atlas_Detail.jsp?atlas_id=15

^2^Available from http://nifti.nimh.nih.gov/pub/dist/src/niftilib/nifti1.h

^3^Available from http://www.fmrib.ox.ac.uk/fsl/; files in directory fsl/data/atlases/JHU/

^4^Available from http://www.fil.ion.ucl.ac.uk/spm/; file canonical/avg152T2.nii

^5^Available from http://www.itksnap.org

^6^Available from http://nitrc.org/projects/mricron/

^7^Available from http://nitrc.org/projects/cmtk/

^8^Available from http://www.slicer.org

^9^http://nitrc.org
